# Influence of Environmental Factors on the Swelling Capacities of Superabsorbent Polymers Used in Concrete

**DOI:** 10.3390/polym12102185

**Published:** 2020-09-24

**Authors:** Andre Jung, Manuel B. Endres, Oliver Weichold

**Affiliations:** Institute of Building Materials Research, RWTH Aachen University, 52062 Aachen, Germany; jung@ibac.rwth-aachen.de (A.J.); endres@ibac.rwth-aachen.de (M.B.E.)

**Keywords:** environmental influence, storage conditions, degree of swelling, sorption isotherms, cementitious materials

## Abstract

Superabsorbent polymers (SAP) are of major interest as materials to control the cement hydration process. The swelling behavior of the SAPs significantly influences the performance of the resulting concrete by slowly releasing polymer-bound water in order to maintain a consistent *w*/*c* value. A round-robin test conducted by the RILEM Technical Committee 260-RSC showed that the same batch of polymer can lead to large deviations in concrete performance and this was assumed to originate in different storage conditions of the SAP. In this contribution the change in the performance of two SAPs, a crosslinked poly(acrylate) and a crosslinked poly(acrylate-*co*-acrylamide), was assessed after ageing in standard climate, at 50 °C, and under UV irradiation. During storage in standard climate or 50 °C, ageing led to dehydration of the SAP, and this subsequently led to a higher water uptake during swelling. By contrast, UV irradiation reduced the water uptake, most likely as a result of photo-crosslinking. Dynamic water vapor sorption experiments indicated a strong dependence of the water uptake on both the ambient humidity and the temperature. As a result, cement mixtures containing SAP must be calculated on the dry mass of the SAP rather than the actual weight on site. A standard procedure of how to pack and handle SAP to be used in concrete is also provided.

## 1. Introduction

The use of polymeric materials in concrete [1,2,3,4] has increased steadily over the last decades. Depending on their structure, polymers can influence the rheological properties [5], increase tensile strength [6] or help control the curing process [7]. Superabsorbent polymers (SAP) [8,9] have gained significant interest as additives in concrete due to their unique properties. These are e.g., the mitigation of autogenous [10] and plastic shrinkage [11] or increasing the freeze–thaw resistance [12,13]. The most prominent types of SAP are anionic, crosslinked polyacrylates. These are best known for their applications in diapers [14], but also as humidity regulating materials [15] in the clothing industry. The degree of swelling of polyacrylates can reach several hundred times their dry mass in demineralized water [16]. This is due to a combination of charge-shielding effects and the ability of water to establish a wide-spread hydrogen-bonded network, which increases the distance between the anionic repeat units of the polyacrylate chains [17]. The sodium ions further increase the degree of swelling by increasing the osmotic pressure within the gel. For ionic SAPs, the degree of swelling strongly depends on the ionic strength *I* [18], calculated according to *I* = ∑_i_ *c*_i__∙_*z*_i_^2^, where *c*_i_ is the molar concentration and *z*_i_ the charge of ion *i*. Additionally, for acrylate-based SAP the degree of swelling also depends on the pH value of the solvent [19,20]. The benefit of using SAPs for cementitious materials arises from the fact that the polymer-bound water can slowly be released into a drying environment [21]. For example, modern ultra-high-performance concrete (UHPC) relies on rather low water-to-cement (*w*/*c*) ratios. With decreasing *w*/*c* ratio the access of the clinker particles to water necessary for completing the hydration process decreases [7]. At the same time, the hydration process is based on exothermic reactions causing the temperature to increase [22]. Both effects have a strong impact on the durability [23] and performance of the hardened concrete. The utilisation of SAPs can solve these problems by acting as an internal on-demand water reservoir [24].

Previous studies of the RILEM Technical Committees 260-RSC on SAPs revealed that samples from the same batch behaved differently when used by different research groups. This non-uniformity was investigated in a round-robin test [25], in which deviations in the degree of swelling of up to 40–50% were reached. Among other influences, deviations in the swelling capacities could e.g., be attributed to climate conditions of the testing region. Based on these findings it was assumed that the storage conditions in combination with environmental factors might substantially influence the behaviour of the SAPs. Apart from temperature cycles, frost, and humidity, another prominent example is UV irradiation. The intensity of solar radiation and with it the exposure to UV varies greatly with the seasons and geographic location. UV irradiation can have a number of effects on polymers, most commonly belated crosslinking [26] and degradation of the polymer network [27,28,29]. The former increases the crosslink density and, as a consequence, reduces the maximum degree of swelling. The latter is accompanied with chain scission, which initially results is higher degrees of swelling, but ultimately destroys the swelling properties. As a consequence, a test protocol was defined to analyse the effect of environmental parameters on the SAP performance. It was decided to store one set of samples at 50 °C, the second set under increased UV radiation, and the third at standard climate (23 °C, 50% RH), in order to compare the observed properties with those of the unaltered as-received samples. Furthermore, water vapour sorption isotherms were recorded at 25 °C and 50 °C in the range of 0 to 90% RH, in order to determine the effect of variations in the air humidity on the amount of polymer-bound water when referring to the dry mass of the SAPs. The results lead to a recommended procedure for handling SAPs, to be used in the preparation of concrete with consistent properties.

## 2. Materials and Methods

The SAP samples were provided by Professor Viktor Mechtcherine (Institute of Materials Science, Technical University of Dresden, Dresden, Germany) and are identical to those used in the aforementioned round-robin test [25]. SAP 1 is reported to be a crosslinked polyacrylate and SAP 2 a poly(acrylate-*co*-acrylamide). Double-distilled water, NaCl (100%), and KOH (95%) were obtained from VWR International GmbH (Darmstadt, Germany). D_2_O (99.9%) and NaOH (≥99.0%) was from Merck KGaA (Darmstadt, Germany), Ca(OH)_2_ (95%) from Alfa Aesar (Kandel, Germany). All chemicals were used as received. The polyester filter bags with a maximum mesh size of 90 µm were bought from Rosin Tech Products (Bethpage, NY, USA). The artificial pore solution was prepared from saturated Ca(OH)_2_ containing 170 mmol/L KOH, 17 mmol/L NaOH and with an ionic strength of *I* ≈ 0.26 mol/L [30,31].

### 2.1. NMR-Spectroscopy

^1^H-NMR spectra were recorded on a Mercury 400 spectrometer (Varian, Palo Alto, CA, USA). The chemical shifts are calculated using the HDO signal at 4.64 ppm as reference. 10 mg of each SAP sample were swollen in 2 mL D_2_O for 1 day in closed vials. Then 0.7 mL of each swollen sample was transferred into an NMR tube.

### 2.2. Thermogravimetric Analysis (TGA)

The TGA measurements were performed on a TGA 4000 system (PerkinElmer, Rodgau, Germany) under a continuous nitrogen flow of 50 mL/min. SAP 1 (5.7088 mg) and SAP 2 (6.0052 mg) were each weighed into ceramic vials. The samples were held at 30 °C for 1 min, and then heated to 150 °C at a rate of 20 K/min, again held isothermally for 6 h and finally cooled to 30 °C at a rate of 10 K/min.

### 2.3. Storage Conditions

Samples of SAP 1 and SAP 2 were subjected to three different storing conditions namely standard climate according to DIN EN ISO 291 2008-08 (23 °C, 50% RH) for four weeks, at 50 °C (17% RH) for 7 d, or under UV irradiation (λ = 300–400 nm, 18 Watt, 23 °C, 50% RH) for 7 d.

### 2.4. Swelling Experiments (Teabag Tests)

Approx. 100 mg of each SAP were weighed into polyester filter bags and submerged in 0.5 L of the desired medium (double-distilled water and NaCl, NaOH, or pore solution) at 22 °C. The mass increase was measured after 3.5 h, when the maximum swelling was reached. Before weighing, the teabags were taken out of the solutions and hung up for 5 min to remove not absorbed liquid. The reported values are an average of three individual measurements.

### 2.5. Water Vapor Sorption

Water vapor sorption measurements were performed on an IGAsorp isothermal gravimetric moisture sorption analyzer (Hiden Analytical, Warrington, UK) with a nitrogen flow rate of 180 mL/min at 25 °C and 50 °C. Double-distilled water was used for humidification in all experiments. Each sample weighing 3–5 mg was first conditioned at 92% RH before starting to record of the desorption isotherm. After that the humidity was lowered to 90% RH. This procedure ensures that the first data point is a true desorption equilibrium. The isothermal measurements were continued by lowering the humidity from 90% RH down to 5% RH in nine steps, followed by zero weight determination at 0% RH and were completed by increasing humidity back to 90% RH in ten steps [32].

## 3. Results and Discussion

**Figure 1:**
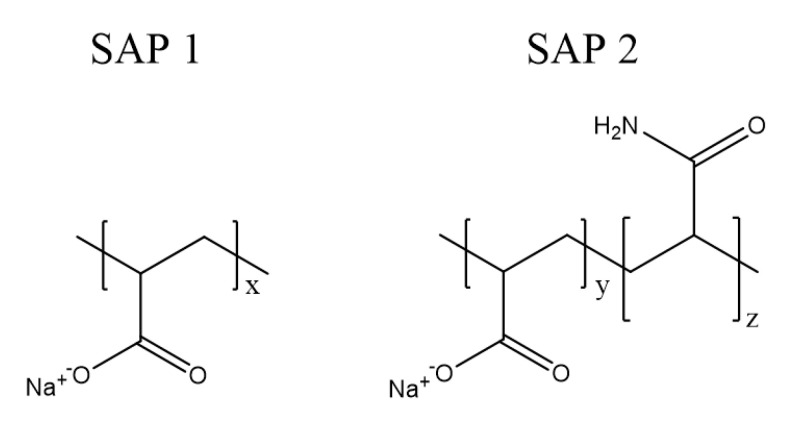
Molecular structure of pure polyacrylate (SAP 1) and poly(acrylate-*co*-acrylamide) (SAP 2).

SAP 1 is an acrylic acid homopolymer that exhibits a high charge density and a rigid structure at full ionization [33]. In contrast, SAP 2 is an acrylic acid copolymer with acrylamide as a non-ionic comonomer (Figure 1). The latter reduces the charge density in the in the network and with it the chain stiffness. Such copolymers have been reported to show increased degrees of swelling and to be less prone to saline media [34,35]. This preferable performance was already shown in the round-robin test [25].

**Figure 2:**
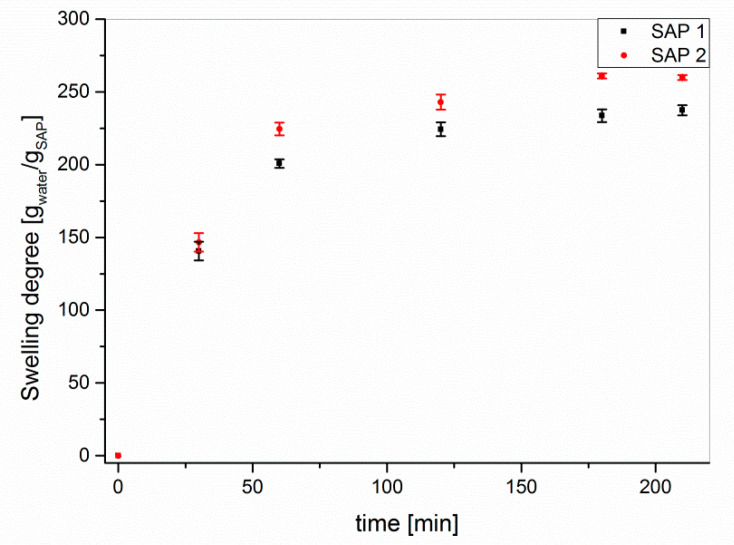
Time-dependent swelling of SAP 1 and SAP 2 as received in double-distilled water.

The swelling experiments were performed under atmospheric pressure. Although the uptake and release of water under load differs from that at atmospheric pressure [27,28], shrinkage cracking in concrete—which should be prevented by the addition of SAP—occurs on the surface, where the SAP are not subject to particularly high hydrostatic pressures [36,37]. The time-dependent swelling experiments of the as-received samples (Figure 2) indicated high degrees of swelling of approx. 250 g/g in double-distilled water with little differences between the two superabsorbent polymers.

**Figure 3:**
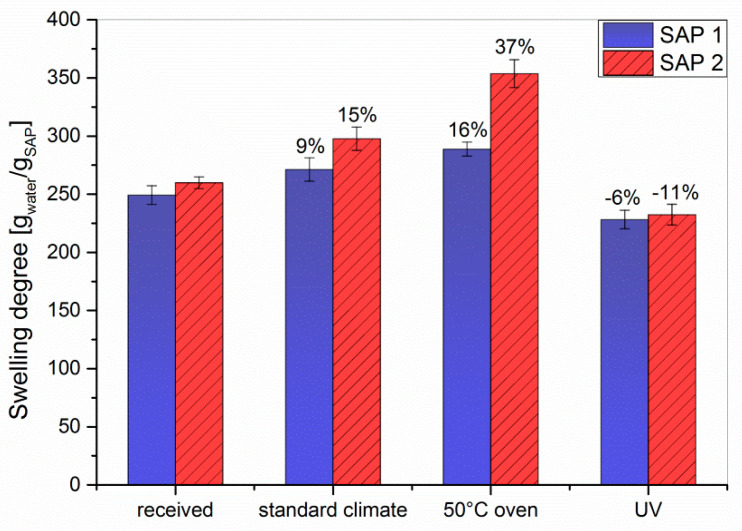
The effect of different storage conditions on the degree of swelling for SAP 1 and SAP 2 in double-distilled water. The values indicate the gain or loss relative to the reference.

The equilibrium appeared to be reached after approx. two hours of swelling. Therefore, the degrees of swelling in the following experiments were determined after 3.5 h to be on the safe side. The storage conditions were deliberately selected not to test for environmental stability of the polymers, but to mimic two climatic conditions in an only slightly exaggerated way. These are hot and dry weather (50 °C at 17% RH for 7 d) and increased solar radiation (λ = 300–400 nm at 18 W for 7 d), which are compared to norm climate according to DIN EN ISO 291 2008-08. Storing both SAPs under standard conditions for four weeks or at 50 °C for 7 d resulted in an increase in the degrees of swelling, which was in both cases larger for SAP 2 than for SAP 1 (Figure 3). The latter is in accordance with the reduced chain stiffness of the SAP 2 copolymer.

**Figure 4:**
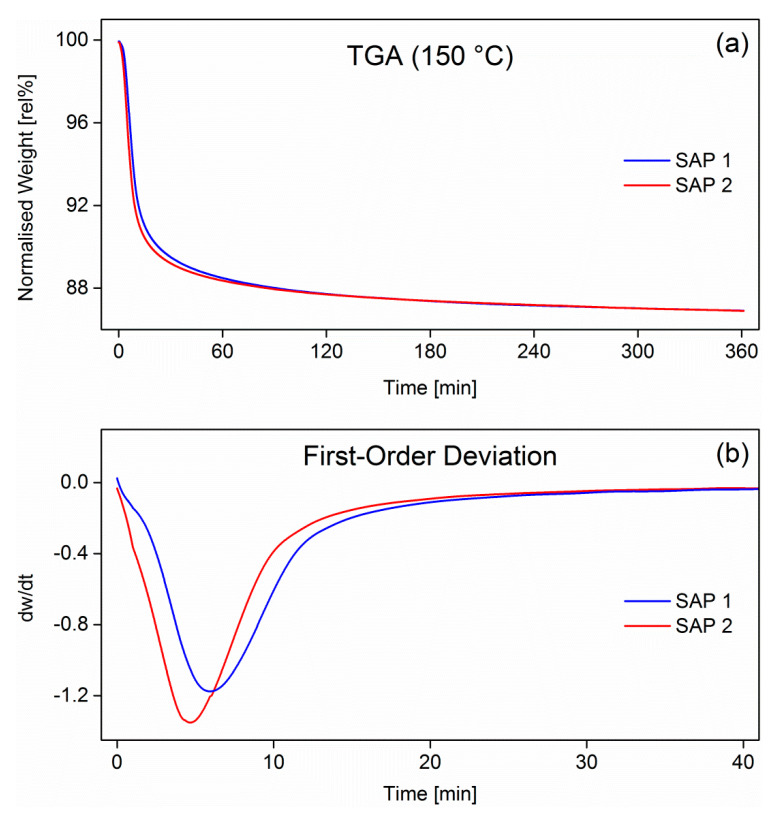
Normalized mass of SAP 1 and SAP 2 measured by TGA (**a**) and the first-order derivative (**b**). Heating rate was 20 K/min. The polymers were held isothermally at 150 °C for 6 h.

One explanation for this behavior could be that the as-received samples were not fully dry and, thus, contained a significant yet undefined amount of water. This was corroborated by isothermal thermogravimetric analyses of the as-received samples (TGA) at 150 °C (Figure 4).

Both samples lost around 12% of their weight in the first 90 min, after which the weight loss levelled off at approximately 14%. However, this did not include the water lost during heating-up, which took approx. 15 min. Although the mass loss vs time curves in Figure 4a appear rather similar, the first-order derivatives in Figure 4b indicate differences in desorption kinetics during the isothermal drying process. SAP 2, which is a poly(acrylate-*co*-acrylamide) copolymer, has more flexible chains compared to the acrylate-based homopolymer, as the charge density of the copolymer backbone is lower due to the presence of the non-ionic acrylamide comonomer [38,39]. Therefore, the desorption of water molecules from the SAP 2 network was faster than in the case of SAP 1, which has a rather rigid structure. This effect can be explained by stronger water-charge and charge-charge interactions [40,41].

**Figure 5:**
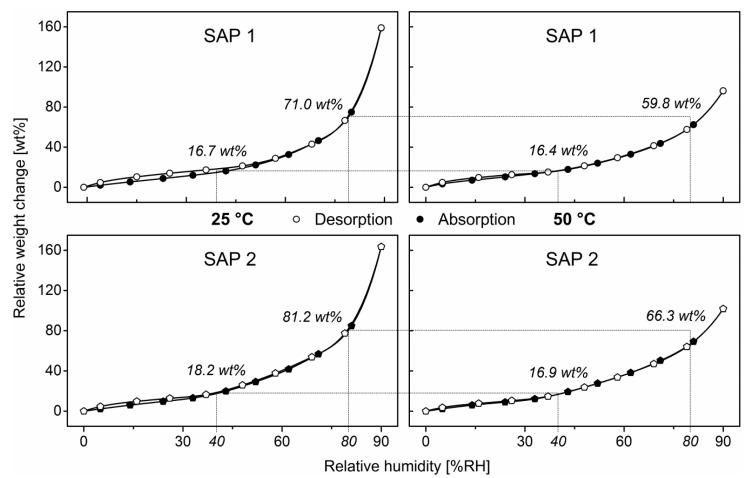
Sorption isotherms of SAP 1 and SAP 2 at 25 °C and 50 °C from dynamic water vapor sorption measurements between 0 and 90% RH with a 180 mL/min humidified nitrogen flow.

To corroborate this, water vapor sorption isotherms in the range of 0 to 90% RH were recorded at 25 °C and 50 °C for both SAPs (Figure 5). All isotherms exhibited convex shapes with an almost linear increase in weight at low humidities followed by a pronounced increase at higher ones and little differences between the two samples. This behavior is typical for BET Type-III sorption isotherms of non-porous materials according to the BDDT model and indicates a moderate affinity between the sorbent (water) and the sorption active substrate (SAP) [41,42,43]. According to this model, it can be said that due to the swift sorption behavior, water molecules are almost instantaneously being absorbed into the bulk of the material without the formation of discrete transient aqueous boundary layers. Both SAPs exhibited only insignificant hysteresis at low humidities, which usually indicates the formation of a rather rigid solvation state [44].

As a result of higher temperatures generally competing with exothermic reactions, the equilibrium between exothermic absorption and endothermic desorption shifted to the right and caused a decrease in the overall amount of polymer-bound water with increasing temperature. Comparing the relative amounts of polymer-bound water at e.g., 40% RH and 80% RH, referring to both rather dry and humid conditions, an increase in temperature from 25 °C to 50 °C did not cause a significant deviation in SAP 1 (Δ ≈ 0.3 wt%). The water concentration of SAP 2 in the sorption equilibrium at 40% RH decreased slightly from 18.2 wt% at 25 °C to 16.9% RH at 50 °C. The temperature-dependence, however, became more obvious at elevated humidities. At 25 °C and 80% RH, SAP 1 contained 71.0 wt% water referring to its dry mass and only 59.8 wt% at 50 °C. SAP 2 was found to be notably more sorption-active at 80% RH with water contents of 81.2 wt% at 25 °C and 66.3 wt% at 50 °C. All in all, SAP 2 absorbs more moisture at both temperatures and all relative humidities than SAP 1, which is again in accordance with the reduced chain stiffness of SAP 2.

As higher temperatures favor the release of polymer-bound water, an increase in temperature applied to a swollen SAP was inevitably accompanied by a significant release of liquid water into the surroundings and vice versa. Thus, when introducing a sizable amount of SAP into cementitious systems, particularly those with low *w*/*c* ratios, the temperature-dependence of the sorption properties could cause an uncontrolled chain reaction in which the heat developed by the cement hydration leads to the release of polymer-bound water, which then might again fuel the cement hydration and evolve even more heat.

**Figure 6:**
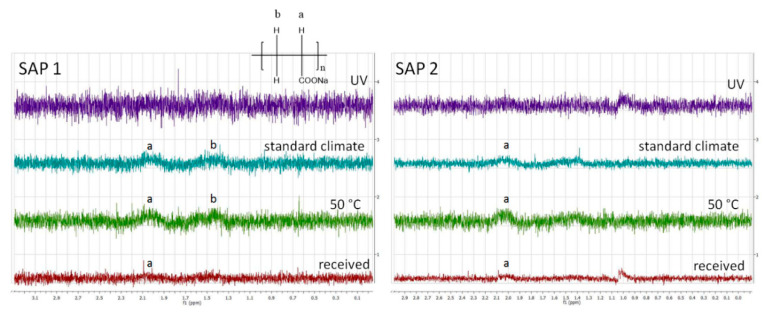
^1^H-NMR spectra of both SAPs swollen in D_2_O after being subjected to the different storage conditions.

In contrast to the influence of temperature and humidity, UV irradiation decreased the degree of swelling of both SAPs (Figure 3). As this exposure was intended to mimic storage in transparent containers rather than to test the UV stability, the irradiation source and time were chosen such that a massive degradation of the polymer structure was avoided. Compared to the as-received samples, the obtained values were 6% lower for SAP 1 and 11% lower for SAP 2. To clarify this behavior, ^1^H-NMR spectra of both SAPs swollen in D_2_O were recorded (Figure 6).

**Figure 7:**
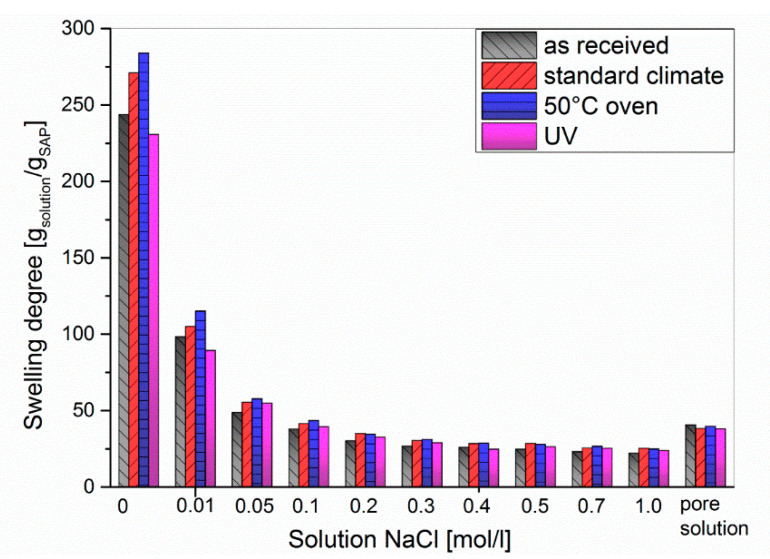
Swelling degree of SAP 1 in sodium chloride solutions.

All spectra showed low signal-to-noise ratios and broad resonances due to insufficient solvation of the polymer network. That means the polymer-polymer interactions are stronger than the polymer-solvent interactions, which leads to a loss of signal. Both as-received SAPs showed two broad resonances of the polymer backbone, one at 2.1 ppm corresponding to the CH−CO_2_Na groups and another at 1.6 ppm originating from the CH_2_ groups [45]. Storing the samples under standard conditions (23 °C, 50% RH) or at 50 °C gave rise to stronger signals. This phenomenon can be explained by desorption of H_2_O upon drying. In contrast, the spectra of the UV-irradiated samples showed hardly any signal at all. This could theoretically be due to UV-induced degradation of the polymer backbone, but appears unlikely as only moderate irradiation conditions were used (vide supra). Another possible explanation might be that irradiation increases the crosslinking density of the polymer, e.g., caused by photooxidation which can spark the formation of radicals in the polymer backbone. Hence, the polymer-polymer interactions are even more pronounced, which consequently reduces the solvent-polymer interactions. As a result of strong hydrogen bonding within the swollen polymer network, the protons of the polymer backbone can no longer be distinguished from their solvating surrounding. A higher crosslinked polymer network would also explain the decreased swelling capacities of the irradiated samples (cf. Figure 3 and Figure 7). To further evaluate the effect of the different storage conditions, the swelling behavior was tested in NaCl solutions as a model system (Figure 7).

**Figure 8:**
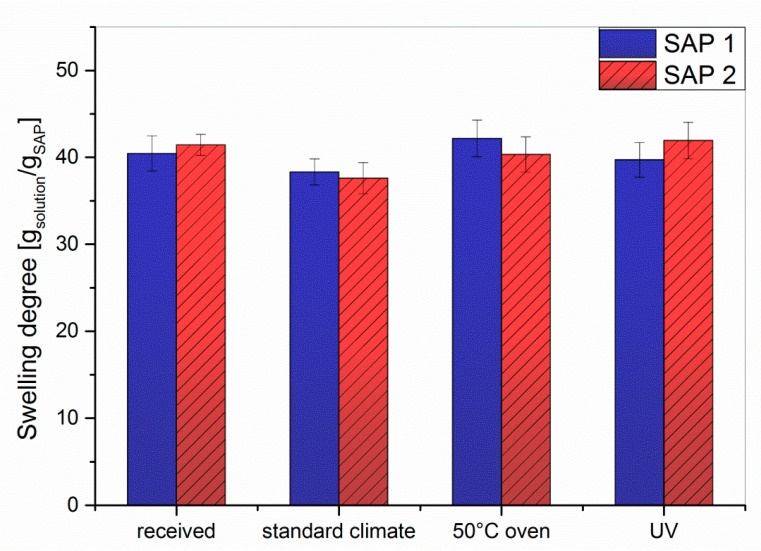
Swelling degree of crosslinked SAP 1 and SAP 2 in artificial pore solution (saturated Ca(OH)_2_, 170 mmol/L KOH, 17 mmol/L NaOH) under different storage conditions.

In general, the degree of swelling decreased when increasing the NaCl concentration up to approx. 0.3 mol/L, after which it remained almost constant at 25 g/g. For NaCl concentrations <0.05 mol/L, the swelling degrees followed the order UV < as-received < standard < 50 °C, which is equal to the behavior in double-distilled water (*c*_NaCl_ = 0, cf. Figure 3). Beyond *c*_NaCl_ = 0.05 mol/L the results differed only to a negligible extent. In order to simulate the conditions in cement paste, the swelling experiments were repeated in artificial pore solution (Figure 8) consisting of Ca(OH)_2_, NaOH, and KOH to obtain a pH of 13.2 [46]. Sulphate, which is sometimes added to artificial pore solutions [47], was not used in this study as we wanted to limit the effects to divalent calcium cations and the pH value. In addition, anions should have little effect on the swelling degree of anionic polyelectrolytes beyond their impact on the ionic strength.

For both samples and under all investigated storage conditions, the degree of swelling in artificial pore solution was approx. 40 g/g. Although the ionic strength of artificial pore solution is *I* ≈ 0.26 mol/L, the swelling here was comparable to that obtained with a 0.1 mol/L NaCl solution (*I* = 0.1 mol/L). At first glance this observation contradicts the theory of SAP swelling, which predicts a lower degree of swelling at higher ionic strengths. In addition, the calcium ions of the artificial pore solution should form insoluble complexes with the acrylate repeat units; this should further reduce the degree of swelling. However, the artificial pore solution has a pH value of 13.2, while the NaCl solutions are neutral. Due to the only moderate acidity of the acrylic acid repeat units, full ionization of both SAPs is only observed at pH values > 10, whereas at the neutral pH of NaCl solutions, the degree of ionization is approx. 40% [48,49,50]. As a result, the fully deprotonated chains in the pore solution experience higher repulsive forces, which leads to stronger swelling. To corroborate this, a swelling experiment was run in 0.1 mol/L NaOH solution, which has the same ionic strength as the 0.1 mol/L NaCl solution (*I* = 0.1), but a pH value of 13. The obtained degrees of swelling in 0.1 M NaOH solution were 51 g/g for SAP 1 and 59 g/g for SAP 2, which is 34% (SAP 1) or 55% (SAP 2) more than in 0.1 M NaCl and 28% (SAP 1) or 48% (SAP 2) more than in artificial pore solution. The comparison between NaOH and pore solution (at a similar pH) also appears to hint to the above mentioned complexation of divalent cations.

These experiments show that the performance of the SAPs is strongly dependent on the salinity and ion composition of the medium, and also the resulting pH value. This becomes particularly clear when comparing the pore solution and distilled water or tap water. Despite its high pH value, the degree of swelling is considerably smaller in pore solution. Hence, mixing SAPs pre-swollen in distilled water into concrete mixtures will lead to an uncontrolled release of large amounts of polymer bound water and, thus, alter the *w*/*c* ratio with negative impacts on the concrete properties.

## 4. Conclusions: Recommended Standard Procedure of How to Pack and Handle SAP for the Application in Cementitious Materials

Based on the study presented here, an easy to use method can now be proposed for reproducible utilization of SAPs as water-retaining ingredients in cementitious systems: since SAPs are sensitive to several environmental conditions, particularly the humidity, the mortar or concrete mixtures containing SAP need to be calculated using the dry weight of the SAP at 0% RH. When stored at ambient climates, SAPs contain an undefined amount of water, which falsifies the true polymer content of the sample. The true dry weight can be easily obtained by freeze drying, thermogravimetric analysis, or calculated from the sorption isotherm, and should be supplied by the SAP manufacturers. However, it is advisable to record a full sorption isotherm for each new type of SAP, in order to understand their behavior under different environmental conditions.

In addition, if SAPs are to be used pre-swollen, this should be done in solutions that closely match the ion composition and pH value of the pore solution of the cement in question, in order to avoid unpredictable shrinking or swelling due to ion exchange between the SAP and the pore solution of the cement. This reduces the potential of concrete batches with inferior quality.
